# Electrochemotherapy and basal cell carcinomas: First-time appraisal of the efficacy of electrochemotherapy on survivorship using FACE-Q

**DOI:** 10.1016/j.jpra.2020.12.004

**Published:** 2020-12-25

**Authors:** Phoebe Lyons, Alison Kennedy, A.James P. Clover

**Affiliations:** a Department of Plastic and Reconstructive Surgery, Cork University Hospital, Cork, Ireland; b Cancer Research @ UCC, Western Gateway Building, University College Cork, Ireland

**Keywords:** Electrochemotherapy, Basal cell carcinoma, FACE-Q, Survivorship

## Abstract

**Introduction:**

The establishment and success of new treatments are significantly influenced by patient satisfaction. Post-operative scarring is an important outcome for patients, and subsequently influences overall satisfaction with treatment. The objective was to measure post-treatment scarring satisfaction using a novel scale, the FACE-Q Skin Cancer Module, to compare electrochemotherapy (ECT) to traditional surgical excision (SE) to demonstrate equivalence of ECT and SE regarding outcome and survivorship.

**Methods and materials:**

This was a multicentre first-time appraisal study of the efficacy of ECT. All patients with facial BCCs treated with either ECT or SE were deemed eligible and subsequently recruited from either a previous clinical trial or outpatient clinics, respectively. Of the 40 participants invited, 25 responses were received. Patient information recorded included age, gender, location and size of BCCs, and time since treatment. Patient outcomes were measured using the FACE-Q Skin Cancer Module.

**Results:**

The ECT and SE groups consisted of 14 and 11 patients, respectively. Mean age was 68 years (M:*F* = 16:9), while mean time since treatment was 4.98 years (range 0.3–9.58 years). Appraisal of scars was significantly higher in the ECT cohort versus SE (*p* = 0.034). Cancer worry was equivalent across both cohorts (*p* = 0.804). According to treatment type, no correlation was detected between time since treatment and both appraisal of scars (ECT *p* = 0.466 and SE *p* = 0.214) and adverse effects (ECT *p* = 0.924 and SE *p* = 0.139).

**Conclusion:**

Based on this study, ECT has superior scar outcomes and overall equivalence to SE. This demonstrates high patient satisfaction for those treated with ECT without any additional cancer worry.

## Introduction

Basal Cell Carcinoma (BCC) is the commonest form of cutaneous malignancy accounting for 62% of all skin cancers.^1^ The annual incidence of BCCs is increasing by approximately 2.5% and 3% for female and male subjects, respectively.^1^Both non-melanoma and melanoma skin cancers affect all socioeconomic classes, ethnic groups and age categories.^2^ Given the increasing incidence and high rates of synchronous lesions, averaging 1.4 per patient^1^, and recurrent lesions, there is an ever-increasing burden on healthcare systems.^3^ Therefore, skin cancer should be considered a global health concern. The destructive nature of BCCs can lead to high rates of physical and psychological morbidity post-treatment as a significant number of lesions occur in both functional and aesthetic areas.^4^^,^^5^ The main risk factor for developing BCCs is intermittent ultraviolet radiation exposure, particularly during childhood and adolescence,^6^ explaining why approximately 80% of all BCCs are located on the head and neck.^2^

The traditional methods of treating BCCs are standard surgical excision (SE) and Mohs micrographic surgery (MMS).^7^ SE is recommended for low-risk lesions and, while SE may be considered in some high-risk cases, MMS is the recommended treatment for high-risk lesions.^7^ Five-year recurrence rates are lowest with MMS for both primary and recurrent BCCs, 1% and 5.6%, respectively.^7^^,^^8^ Both standard SE and MMS have been shown in randomised control trials to have similar aesthetic outcomes, despite the fact that MMS conserves more tissue resulting in smaller defects.^7^ Other non-surgical treatment options include cryotherapy, topical therapies, such as imiquimod, radiation therapy, photodynamic therapy,^7^^,^^8^ and most recently electrochemotherapy (ECT).^9^

ECT is a locally ablative tumour treatment that has recently been approved for many cutaneous tumours and skin metastases.^10^ We have recently published the first prospective randomised control trial comparing ECT against the standard of care SE showing excellent efficacy of the treatment in the control of primary BCC and showing a durable response of this treatment on over 90% of lesions treated after 5 years of follow up.^11^ The principle of ECT is based on the local application of electrical pulses to increase cell permeability allowing normally poor or impermeable chemotherapeutic drugs to enter the tumour cells, without affecting healthy tissue surrounding the lesion.^10^^,^^12^ Typically, bleomycin or cisplatin are administered either locally or intravenously prior to the application of the electrode, while bleomycin is by far the most commonly used.^10^ ECT is particularly useful for patients with significant co-morbidities who are unsuitable for other treatments, those who have a high burden of disease, or those who have an increased risk of functional or aesthetic impairment due to location.^13^

Given the increasing incidence yet low mortality rates of BCCs, survivorship post-treatment is very important. Survivorship includes both physical and psychosocial side effects following treatment, which can be disabling and often permanent.^14^ Furthermore, these outcomes can place a heavy burden on healthcare services.^14^ Of these post-treatment side-effects, scarring can significantly affect patients’ quality of life and self-perception, leading to psychological morbidity^15^ and it is often underestimated by medical professionals.^16^ In turn, this can impact on overall patient satisfaction regarding treatment and care.^5^ Accordingly, improving old and developing acceptable new treatments are essential to improve physical outcomes, which in turn may aid in improving psychosocial outcomes and, therefore, survivorship. However, the objective measurement of patient outcomes following BCC treatment has previously been difficult due to a lack of validated patient-reported outcome measures (PROMs).^5^ PROMs are essential as patient perception of successful outcomes, including satisfaction with scarring and appearance can often differ from the surgeon's opinion.^5^ The FACE-Q Skin Cancer Module, developed from the original FACE-Q,^17^, ^18^, ^19^ is a recently validated, novel PROM that determines outcomes specifically important to facial skin cancer surgery, such as cancer worry and overall scarring and appearance.^5^ While there has been a significant increase in the number of PROMs, there remains a paucity of outcome data for ECT treatment of BCCs.

The aim of this study was to objectively and comprehensively compare SE and ECT outcomes using this validated, novel scale. The objectives included determining post-treatment scarring satisfaction to allow a comparison between treatments; and demonstrating equivalence between traditional SE and ECT regarding outcome and survivorship using the new FACE-Q Skin Cancer Module.

## Materials and methods

### Study design

This is a first-time appraisal study of the efficacy of ECT that was undertaken in two centres: Cork University Hospital, Cork, Ireland, and The South Infirmary Victoria University Hospital, Cork, Ireland. All patients aged 18 or over with facial BCCs who underwent either surgical excision or ECT from January 2010 to July 2018 were invited to take part. Ethical approval was sought from the Clinical Research Ethics Committee of Cork Teaching Hospitals.

### Participants

Two patient groups were identified based on treatment received: SE or ECT. Participants in the SE cohort were identified when attending the plastic surgery. Outpatients’ department and were subsequently recruited. Patients in the ECT cohort were recruited, in part, from a previous clinical trial of ECT on BCCs carried out by the plastic surgeon eight years previously. This cohort comprised of 55 patients in total, of which 30 were eligible to participate.

### Study measures

The FACE-Q Skin Cancer Module questionnaire (FACE-Q™ Memorial Sloan Kettering Cancer Centre) was used to objectively measure patient outcome and satisfaction post- operatively. This module is comprised of four individual scales: Satisfaction with Facial Appearance, Cancer Worry, Psychosocial Distress, Appraisal of Scars; and two checklists: Adverse Effects, and Sun Protection Behaviour (Appendix 1). In addition to the FACE-Q scales and checklists, other variables collected from the patients were age, gender, location of and size of lesion, time since treatment, and any history of previous BCCs.

### Data analysis

Data were analysed using GraphPad Prism 8 (GraphPad Software, Inc., California). Descriptive statistics were ascertained, while significance was set at *p*<0.05. The Shapiro-Wilk test was applied to test for normality. All data sets were non-normally distributed; therefore, the non-parametric Mann-Whitney U test was applied to assess treatment type and outcome. Correlation between outcome variables and patient variables were analysed using the Pearson's correlation coefficient. The relationship between time since treatment and adverse effects, and the appraisal of scars was determined using the linear regression analysis.

## Results

### Patient demographics

Of the 40 patients invited to participate, a total of 25 patients (16 men and nine women) completed the FACE-Q questionnaire. The ECT and SE cohorts consisted of 14 and 11 patients, respectively. The average age of participants was 68.2 years (range, 52–87 years). The overall mean time since treatment was 4.98 years (range 0.3–9.58 years), while time since treatment was significantly longer in the ECT group as compared to the SE cohort (*p*<0.0001). Patient characteristics are described in detail in Table 1. The Shapiro-Wilk test for normality demonstrated non-normal distribution for all outcome measures. Therefore, the non-parametric Mann-Whitney U test and the Pearson's correlation coefficient were utilised.

**Table 1:** Patient demographics.

	ECT	SE	Overall
No. of Participants	14	11	25
Male	7	9	16
Female	7	2	9
Age (mean +/- SD)	70 (+/- 9.01)	65.7 (+/- 9.03)	68.2 (+/- 9.1)
Time since Treatment (yrs) (mean +/- SD)	7.26 (+/- 0.82)	2.07 (+/- 2.62)	4.98 (+/- 3.19)
Size of Lesion (cm) (mean +/- SD)	1.29 (+/- 0.72)	1.39 (+/- 1.01)	1.34 (+/- 0.84)

### Appraisal of scars

**Fig. 1:**
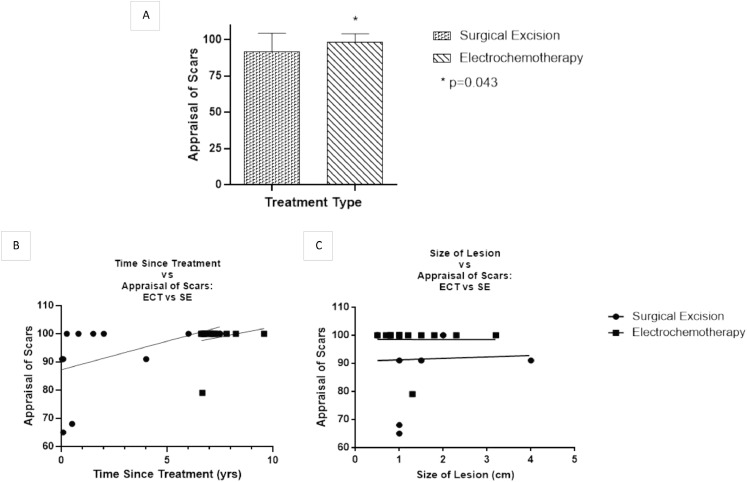
A) Mann–Whitney U test: a significantly higher appraisal of scars in the ECT cohort. B) Linear regression: time since treatment and appraisal of scars. C) Linear regression: size of lesion and appraisal of scars.

Mean appraisal of scars was 91.45 in the SE cohort (95% CI=82.72 and 100.18) and 98.5 in the ECT cohort (95% CI=95.26 and 101.74), where higher values reflect higher satisfaction with scar outcome. ECT had significantly higher satisfaction with scars than SE (*p* = 0.043) (Fig. 1A). When divided into treatment type, Pearson's correlation coefficient showed no correlation between the appraisal of scars and time since treatment (SE: *r* = 0.407, *p* = 0.214 and ECT: *r* = 0.212, *p* = 0.466) or lesion size (SE: *r* = 0.04, *p* = 0.907 and ECT: *r*=−0.002, *p* = 0.992). The linear regression analysis also showed no significant relationship between time since treatment and appraisal of scars (SE: r^2^=0.166 and ECT: r^2^=0.045) (Fig. 1B and 1C, respectively).

### Adverse effects

**Fig. 2:**
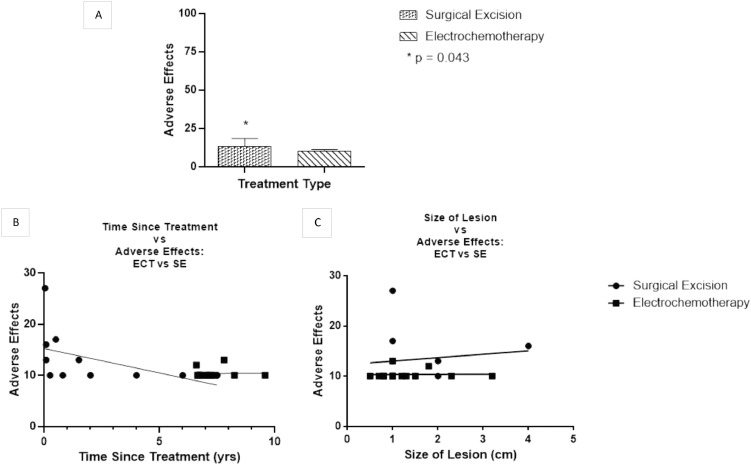
A) Mann–Whitney U test: Significantly more adverse effects reported in the SE cohort. B) Linear regression: time since treatment and adverse effects. C) Linear regression: size of lesion and adverse effects.

Mean score for adverse effects was 13.27 in the SE cohort (95% CI=9.75 and 16.79) and 10.36 in ECT cohort (95% CI=9.82 and 10.89), where higher values represent more adverse effects experienced in the previous week. SE patients reported a significantly higher number of adverse effects than ECT patients (*p* = 0.043) (Fig. 2A). When divided into treatment type, no correlation was detected between adverse effects and time since treatment (SE: *r*=−0.476, *p* = 0.139 and ECT: *r* = 0.0286, *p* = 0.923) or lesion size (SE: *r* = 0.132, *p* = 0.699 and ECT: *r* = 0.0156, *p* = 0.9579). Additionally, linear regression failed to show any significant relationship with time since treatment and adverse effects (SE: r^2^=0.226 and ECT: r^2^=0.001) (Fig. 2B and C, respectively).

### Cancer worry

**Fig. 3:**
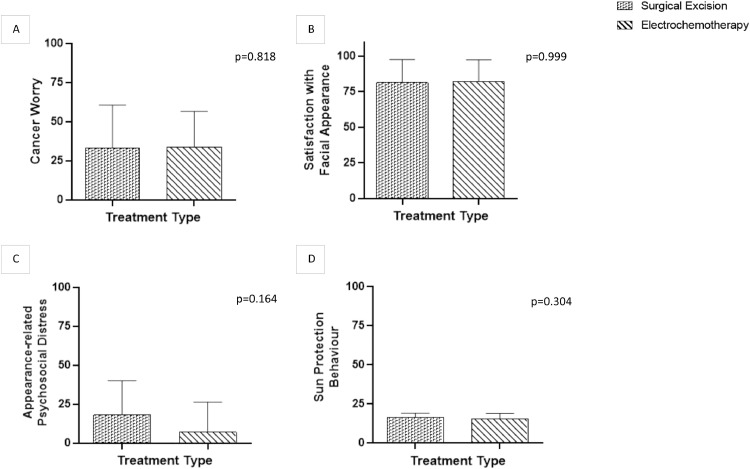
Mann–Whitney U test showing no significance between treatment groups and A) Cancer worry, B) Satisfaction with facial appearance, C) Appearance-related psychosocial distress and D) Sun protection behaviour.

Mean score for cancer worry was 33.27 in the SE cohort (95% CI=14.85 and 51.70) and 33.79 in the ECT cohort (95% CI=20.59 and 46.98), higher values inferred higher levels of worry regarding their cancer. When tested, no significant difference was detected between treatment groups (*p* = 0.804) (Fig. 3A). Similarly, no correlation was detected between cancer worry, and time since treatment (SE: *r*=−0.583, *p* = 0.06 and ECT: *r* = 0.341, *p* = 0.233) or lesion size (SE: *r*=−0.159, *p* = 0.641 and ECT: *r* = 0.319, *p* = 0.266). Furthermore, linear regression found no significant relationship with either cohort (ECT: r^2^=0.34 and SE: r^2^=0.116).

### Satisfaction with facial appearance

Mean score for satisfaction with facial appearance was 81.36 in the SE cohort (95% CI=70.34 and 92.38) and 82.14 in the ECT cohort (95% CI=73.33 and 90.95), where higher values show higher satisfaction. Analysis showed no significant difference between SE and ECT cohorts (*p* = 0.999) (Fig. 3B). Furthermore, no correlation was observed between satisfaction with facial appearance and both time since treatment (SE: *r* = 0.154, *p* = 0.651; ECT: *r* = 0.349, *p* = 0.222) and size of lesion (SE: *r* = 0.212, *p* = 0.532; ECT: *r*=−0.471, *p* = 0.089), when divided into treatment type. Similarly, linear regression detected no significant relationship with time since treatment (SE: r^2^=0.024 and ECT: r^2^=0.122) or size of lesion (SE: r^2^=0.045 and ECT: r^2^=0.222).

### Appearance-related psychosocial distress

Mean score for appearance-related psychosocial distress was 18.00 in the SE cohort (95% CI=3.11 and 32.89) and 17.14 in the ECT cohort (95% CI=−4.02 and 18.31), where higher scores represent higher levels of distress. The analysis failed to show a significance between patient groups (*p* = 0.164) (Fig. 3C). When analysed according to treatment type, no correlation was detected between appearance-related psychosocial distress, and either time since treatment (SE: *r*=- 0.203, *p* = 0.55 and ECT: *r*=−0.01, *p* = 0.978) or lesion size (SE: *r*=−0.21, *p* = 0.535 and ECT: *r* = 0.032, *p* = 0.915). Furthermore, linear regression failed to show any relationship between outcome and time since treatment (SE: r^2^=0.041; ECT: r^2^=0.0001) or size of lesion (SE: r^2^=0.044; ECT: r^2^=0.001).

### Sun protection behaviour

Mean value for sun protection behaviour was 16.73 in the SE cohort (95% CI=15.11 and 18.35) and 15.5 in the ECT cohort (95% CI=13.48 and 17.52), with higher scores indicating more protective behaviours. Significance was not detected between patient cohorts (*p* = 0.304) (Fig. 3D). When divided into treatment type, correlation was detected in the SE cohort between sun protection behaviour and lesion size (SE: *r*=−0.711 and *p* = 0.014). However, this was not detected in the ECT cohort (ECT: *r* = 0.175 and *p* = 0.55). Similarly, a correlation was not observed in either patient groups regarding sun protection behaviour and time since treatment (SE: *r*=−0.003, *p* = 0.993 and ECT: *r* = 0.49, *p* = 0.074). Linear regression showed there to be a significantly negative relationship between lesion size and sun protection behaviours in the SE group (r^2^=0.506), but not in the ECT cohort (r^2^=0.031).

## Discussion

The importance of survivorship has been brought to the forefront of clinical practice as a result of the increasing understanding of its importance to patients. Scarring and adverse effects following treatment for low-recurrence, low-mortality cancers such as BCCs^1^ are important outcomes that can have a significant impact on patient quality of life and satisfaction with treatment.^20^ As a result, PROMs including the FACE-Q Skin Cancer Module are an important tool in improving treatment of such cancers on cosmetically sensitive areas. The FACE-Q Skin Cancer Module has been validated as a tool to objectively measure survivorship post-treatment through measuring satisfaction with scars and facial appearance, adverse effects, and the feeling of comprehensive treatment in the form of cancer worry.^5^^,^^21^

Satisfaction with scarring is an important outcome for patients and can significantly affect patients’ overall satisfaction with treatment.^5^ Amongst our patients, ECT reported higher satisfaction with scarring than that of SE, while the SE patients reported significantly more adverse effects. Given the large differences in time since treatment between ECT and SE cohorts (mean difference = 5.19 years), time could account for the significances achieved. However, linear regression suggests that time is not a confounding factor for either treatment, further implying that superior scar outcomes and reduced adverse effects were achieved with ECT.

Given the low mortality rates for BCCs, reducing scarring should be an important priority.^1^ However, some lesions, because of size, number or location, are not possible to remove without significant disfigurement. This is where methods, in particular ECT, are proving beneficial^11^^,^^13^; yet, it is not widely available for such patients. Increasing ECT accessibility could improve patient outcomes and, therefore, survivorship. This would reduce the burden on healthcare services, including support services, as a result of function impairment from scarring, but also psychological aspects, including societal withdrawal and psychosocial distress.^17^ Similarly, adverse effects such as pain, numbness, tingling or itchiness can seriously affect patients’ quality of life for weeks to months post-treatment, increasing both psychological and physical morbidity.^5^^,^^14^ ECT showed superiority regarding two physical yet subjective aspects of survivorship, further suggesting that ECT should be increasingly considered as part of the treatment toolbox for BCCs.

Of particular importance amongst the non-significant results, cancer worry was near equivalent amongst the two treatment groups. Post-treatment worry about cancer recurrence is a notable concern for a considerable number of patients.^14^ Additionally, patients treated for one cancer often have an increased risk of developing the malignancy elsewhere, because of either a genetic predisposition or due to the same causative environmental exposure,^14^ for example, Gorlin's syndrome^22^ and ultraviolet radiation exposure,^3^ respectively. The equivalence of cancer worry between both groups demonstrates equal patient confidence in treatment efficacy.

The results interestingly showed a negative relationship between fewer sun protection behaviours and larger lesion sizes, but only in the SE patient group. One patient in the ECT group suffered for many years from the Gorlin syndrome, also known as naevoid BCC syndrome, and despite having the largest lesion size, the patient scored very high on the sun protection behaviour checklist. Outliers such as this patient may account for the lack of significance in the ECT cohort. However, given that UV exposure, particularly prolonged intermittent exposure in adolescence, is a well-known considerable risk factor for developing BCCs^2^^,^^6^^,^^23^; it is not surprising that there is a negative relationship between lesion size and sun protection behaviours. In addition, the Irish population in general have several other risk factors, including fair skin, red hair and light eye colour,^2^^,^^6^^,^^23^ possibly further accounting for the association detected.

This study is the first of its kind to assess the use of the novel PROM, the FACE-Q Skin Cancer Module for ECT. The results from this study could be generalisable to the Irish population, and given the prevalence of BCCs in Ireland, this information could be very valuable to clinicians when they suggest treatment for BCCs. However, future studies are still required to fully corroborate the results found in this patient group. Another strength lies with the inclusion of two centres for patient recruitment to reduce selection bias.

The first and main limitation of this study is the sample size. Because of the small sample size of each cohort, definitive conclusions cannot be drawn from the data as they may overestimate the associations detected. A larger sample size would increase the power of the study and ultimately the results. Secondly, there is a possibility of selection bias as 100% of patients approached in the outpatients’ department completed the questionnaire, while this was notably lower with questionnaires issued by post. Thirdly, equal number of male and female patients in each treatment arm were not achieved, with more male than female patients. As females are often more affected by changes in facial appearance,^24^ the outcomes measuring such may underestimate the effect in the female population. Lastly, time since treatment was notably different between treatment groups and should be controlled for in future studies.

To definitively demonstrate equivalence between treatments or, potentially, the superiority of ECT, a large blinded randomised control trial should ideally be undertaken. This could allow for a wider availability of ECT for skin cancer patients, particularly those with significant co- morbidities or increased risk of scarring due to size, location or number of lesions.

## Conclusion

This study, however, achieves its two main aims: it shows the benefit and worth of the FACE- Q Skin Cancer Module in assessing outcomes after the treatment of BCCs, while also demonstrating equivalence between ECT and SE regarding outcomes. This PROM shows merit in describing patient satisfaction after treatment by incorporating a score acknowledging the impact of scarring and facial appearance offset against cancer worry. Moreover, this is the first objective PROM that assesses the impact and efficacy of ECT as a treatment for BCCs. BCCs are successfully and durably treated by ECT and here, we show that this results in equivalent cancer worry. This demonstrates patient satisfaction with treatment in addition to improved satisfaction with scarring, suggesting a potential benefit of ECT in aesthetically sensitive locations.

## Declaration of Competing Interest

None.
